# A Treatise on the Nature and Treatment of Scrofula

**Published:** 1822-09-01

**Authors:** 


*A Treatise on the Nature and Treatment of Scrophula, &c.*
By Eusebius Arthur Lloyd, &c.

[Second Analytical Article, concluded from page 174 of this Volume.]

In our first analytical article we were unable to comprehend the important subjects of osseous, articular, and visceral scrophula; to these, therefore, we now proceed.

1. *Osseous Scrophula.* Mr. Lloyd observes, that bones are liable not only to inflammation and all its consequences, in common with other parts, but also to *specific diseases*, where a disposing state of constitution exists—for instance, syphilis and scrophula. Each of these last diseases, however, appears to have a favourite osseous seat—thus syphilis attacks the more hard and compact bones, seldom invading the cancellous structure and joints. Scrophula, on the other hand, usually affects the softer and more spongy bony structures, as the heads of the cylindrical bones, the bones of the carpi, tarsi, and vertebræ. The cancellous structure is, also, involved in scrophulous disorganization, as many preparations in Mr. Langstaff's museum testify.

The true scrophulous disease of bone, Mr. L. thinks, always commences in the cellular or cancellous structure—the vascularity being merely increased at first, then the whole texture becomes altered, the earthy matter absorbed, the bone softer than in health. Subsequently, the cancellous structure itself becomes absorbed—the cancelli are filled with a yellow caseous matter, or transparent yellow fluid. How long the bones may remain in this incipient state of disease, it is difficult to say; but the next effect appears to be a thickening and swelling of the external parts, from a deposition of gelatinous fluid into the cellular substance, and round the tendons and ligaments of the joint. Although this thickening often goes to a great extent, and continues long in an indolent state, yet the true character is at length developed, and more active inflammation comes on. The cartilages ulcerate or are absorbed—the synovial membrane inflames—matter forms—and the whole joint becomes included in the disease. In scrophula, of whatever structure, the vascularity, our author thinks, is at first increased, but ultimately diminished, as is proved by injecting parts in the incipient and more advanced stages of the disease. Mr. Lloyd combats the idea, that the morbid alteration of structure in scrophulous bones is the product of an inflammatory process going forward in all stages of the complaint.

"I may observe here," says he, "that it is a great mistake to consider the scrophulous matter, such as is found in the bones, the glands, or what scrophulous tubercles are formed of, as a new growth, or as a species of tumour, for really *it is a mere secretion* or deposition of unorganized matter. Its being a secretion too, is an argument against an inflammatory state of the bone continuing after the deposition has taken place, for the common and invariable effect of inflammation, is to put a stop to all secretion in whatever part it attacks, as we often witness in the liver, kidneys, and many other parts of the body, and the secretion comes on, or is restored, only as the inflammation subsides." 125.

There is some loose and some erroneous reasoning in the foregoing passage. Although we are not disposed to deny, that the scrophulous matter may be a *secretion*, yet we do not agree with Mr. Lloyd, that inflammation cannot have any share in this process. Does inflammation of the mucous membrane of the nose, trachea, urethra, &c. check secretion there?—Did Mr. Lloyd ever open a patient that died of peritoneal inflammation, and find these evidences of checked secretion? In fact, there is not a more common morbid phenomenon, than increase and depravation of secretion, during an inflammatory process in a structure. In some of the larger glandular organs, indeed, as the kidneys, liver, &c. we know that a *high range* of inflammation is incompatible with secretion; but we are much inclined to think, that lower grades of the same disease, and particularly that state called *irritation*, very frequently increases and deteriorates the secreting functions even of those large viscera.

The ulceration of the cartilages of joints, Mr. Lloyd observes, appears clearly to depend on the diseased state of the bone, or on inflammation of the synovial membrane, rather than on any morbid condition of the cartilage itself. How rapidly the cartilages will sometimes ulcerate, when the synovial membrane is inflamed, every surgeon conversant with the morbid anatomy of the joints, well knows. When an abscess forms in the bone itself, the matter sometimes makes its way through the cartilage into the cavity of the joint, occasioning very severe symptoms. At other times, the matter makes its way to the surface below the cartilage and the attachment of the capsule, so that the cartilage remains entire. Under these circumstances, abscesses may form in the surrounding parts, as when the whole joint is diseased. Our author denies that necrosis and spina ventosa can ever be the consequence of scrophula, though he has heard a public lecturer assert that they are. Scrophula, he remarks, comes on gradually, and without pain or acute inflammation; while, on the contrary, necrosis comes on suddenly and rapidly, and is often attended with most violent pain and acute inflammation. In scrophula, there is a continual absorption of bony matter, while, in necrosis, the original bone being dead, there is an immediate, and often an excessive deposition of new bone. Spina ventosa, in its modern acceptation, cannot, he thinks, be considered as scrophulous, though, in former times it might, as it then included all diseases of the bones commencing in their interior.

Like other diseases dependent on a scrophulous diathesis, the affection of the bones seldom occurs, except in early life —although there is no period entirely exempt from the disease where the scrophulous diathesis is strong. From our author's diagnostic or distinctive symptoms of the disease, we shall cull the following particulars.

In the early stage, when there is simply absorption of the earth, and a morbid deposition in the cancelli of the bone, the change takes place so insidiously and imperceptibly, that it is next to impossible to detect it. If there be any symptom produced, it is merely a sense of slight weakness in the part, which is generally attributed to constitutional debility. The first decided symptom of disease going on in the articulating extremities of a bone, is an occasional deep-seated, dull, heavy pain unattended by swelling, and not increased by motion; but if it be the hip, knee, or ankle joint, which is affected, the pain is somewhat increased by the compression of long standing or walking. This state often continues for many months; but generally the pain gradually increases—after exercise the joint swells, but subsides at night—at length the soft parts swell permanently, but not extending to the whole joint as in other diseases. In the knee it often commences on either side, just behind the condyles, so that the joint appears wider, and more spread out than it naturally is—the swelling gradually increasing and affecting the whole joint, feeling elastic, but at the same time firm, as if the heads of the bones were enlarged. The superincumbent skin is tense, smooth, and transparent, displaying conspicuously some large blue veins on the surface. Even in this stage, though there is more pain than in the first, yet, except occasionally, it is rather a continual uneasiness than actual pain of the joint.

As the disease advances the inflammatory condition increases, together with the pain, and a constant feeling of heat in the part. The constitution now sympathizes with the local irritation. An abscess often occurs at this period from an accidental circumstance, in some part of the swelling, which bursts, and continues discharging for a short time, when it heals—the parts sometimes continuing for months, or even for years, without the formation of fresh abscesses.

When, however, the third, or suppurative stage is fairly established, it is very common to have a succession of external abscesses not communicating with the cavity of the joint, which burst and readily heal, or leave sinuses which often continue for a long time to discharge a sero-purulent matter. In the progress of the case, however, the articular cartilages ulcerate, and matter forms in the cavity of the joint—the matter sometimes issuing at a prominent point, but oftener insinuating itself in every direction above and below, and discharging itself at a considerable distance from the joint. It is needless to say, that hectic fever is but too generally established under this state of things.

When the ankle is affected, the swelling generally commences on the anterior part, and is from the first diffused; it extends to the sole of the foot and metatarsus; the foot becomes almost round, and is immensely enlarged; the muscles, from disease, lose their power; the toes remain motionless, appear smaller than is natural, and pale as if they were dead; suppuration ensues, and its results are modified by the effects which medicine or the reactions of nature produce.

When parts of the fingers and toes are affected, they often suppurate, and exfoliation of bone, to a greater or less extent, succeeds. A joint, in such circumstances, may appear to be anchylosed, and yet its motion be ultimately recovered. When one end or the whole of the phalanx dies and comes away, regeneration never takes place, and the finger or toe is permanently shortened.

The shoulder experiences a less degree of enlargement in scrophula than any other joint; at first the swelling is most obvious about the coracoid process; at a latter period, the axilla exhibits the greatest fulness. Abscesses are not frequent in its external parts; and, when matter forms in the articular cavity, it makes its way to the surface from under the posterior margin of the deltoid muscle.

Scrophula of the elbow-joint generally commences at the bottom of the inner condyle of the humerus, or by the swelling of a gland just above that projection. There is always some degree of enlargement between the condyles and olecranon, and at the radial and ulnar connexion; but the principal tumour is in the bend of the arm, where it is often so considerable as to make the fore-arm, when bent, appear much shortened. The implicated parts are seldom so hard as those of the knee, carpus, and tarsus, when similarly diseased; they possess some degree of firmness and elasticity; the skin is pale and shining; and the superficial veins become dilated.

Mr. Lloyd's manner of accounting for the muscular atrophy of the limb which accompanies many forms of disease in the joints is rational and consistent with the laws of vital action.

"The true explanation," says he, p. 147, "of the wasting of particular muscles is, that their action giving pain in the joint, or at least some uneasiness, they cease to act, and consequently waste away, which every physiologist well knows is an invariable consequence when parts become useless, or their action occasions uneasiness. When the shoulder joint is diseased, we ought not to be surprised that some muscles of the humerus should waste away in a greater degree than others, as the action of some may, from necessity, have been kept up, while others may not have acted at all; or the slightest action of some may cause pain, while others may, and must be able to act to a considerable degree, without exciting the slightest pain. When it is the knee or hip-joint that is affected, it is common, in the advanced stage, for the limb to become œdematous."

It is a mistake to say that bones *cannot* become enlarged unless by *external* deposition: there are several preparations in existence which prove that they may be actually expanded by *interstitial* deposition.

*Treatment.* The constitutional treatment of the scrophulous habit, and of the disorder of health consequent on it, has already been described. It is here to be employed with assiduity and circumspection. So long as the vital actions remain unimproved, the local treatment should not embrace more objects than these—avoiding all sources of pain, irritation, and inflammatory excitement; securing to the affected member the greatest degree of rest possible; and such like palliative measures. When the shoulder-joint is the seat of scrophulous lesions, the arm must be supported by a sling, fastened to the patient's side, so as to prevent motion by elevation and depression. When the elbow or wrist-joint is diseased, it will be sufficient to keep the whole fore-arm in a state of semiflexion, suspended in a sling to which a splint has been adapted. In this affection of the wrist an external splint is not requisite; but it is of great importance to keep the fingers in a proper degree of flexure. When the knee is diseased, the limb should be kept perfectly straight and steady, either by two short splints, one on each side, extending a few inches above and below the joint, or by one long splint reaching from the trochanter major to the foot; the patient, at the same time, being confined to bed. When the ankle-joint or foot is the part affected, confinement to bed and the use of a splint are indispensable. In such cases the outer splint used for a fractured leg answers every purpose.

When it is applicable, moderate exercise should be enjoined. Inflammation and its sequences may be obviated by the judicious employment of leeches; abscesses are to be opened with the lancet; when swelling and sinuses remain, and are not accompanied with irritation, setons and issues are sometimes efficacious in removing them; when there are no open sinuses, blisters or friction with tartar-emetic ointment and moderate compression with proper bandages, will be profitable.

With the detail of eighteen cases illustrative of these practical doctrines, Mr. L. concludes this very interesting section. They are highly instructive; but our limits forbid our transcribing more than one: it is a case of scrophulous shoulder-joint.

" A boy, nine years of age, was, three years before Mr. L. saw him, attacked with slight occasional pain in his right shoulder attended with weakness and an indisposition to use it. He was, at the same time, in a very delicate state of health, had costive bowels, and very bad appetite, and was also affected with scrophulous ophthalmia. There was not much difference between the size of the two limbs, but the muscles about the humerus and scapula appeared to have somewhat wasted away, and were very flaccid. The patient was now taken to one of the most celebrated London surgeons, who considered it a case of paralysis, and consequently ordered it to be moved about as much as possible, and to be electrified twice a week: at the same time he prescribed for him some tonics. This plan of treatment was persisted in for nearly three months, but then, as the child was much worse in every respect, he was taken to another surgeon, who ordered the arm to be kept in a sling, yet at the same time the whole limb to be well rubbed, night and morning, and tonic medicines to be continued. He persisted in this treatment for several months, as the constant rest rendered the pain in the arm much less severe than it had been while he was pursuing the former plan of treatment. However, as there was no evident amendment in the disease, and as the joint was observed to swell, he now became a patient of mine.

" At this time, the head of the humerus appeared enlarged, and the edge of the glenoid cavity thickened, as did the coracoid process of the scapula. All the muscles arising from the scapula, and attached to the humerus, and indeed all the muscles connected with that bone, had very much diminished in size; but least of all the deltoid. There were apparently very few fibres of the biceps remaining, but these were sufficiently strong to bend the fore-arm with considerable force. Among the other muscles connected with the humerus were the latissimus dorsi and pectoralis major.

" There was considerable motion remaining in the joint as the arm might be rotated and elevated to a certain degree, but beyond that all motion was performed by the elevation or depression of the scapula on the trunk. The muscles of the fore-arm remained of their natural size, and retained their usual power, as the right hand could be kept firmly clenched for as long a period as the left. The pain in the joint was not severe, and but slightly aggravated by motion, except where an attempt was made to move the humerus beyond the contracted sphere that has been described before, the pain however was generally worse during the night than in the day. His general health was very bad; he was very weak; his appetite was very indifferent; his tongue was white and furred; his bowels were costive, and his pulse seldom less than ninety. From the whole state of the case, I pronounced the disease a scrophulous affection of the shoulder joint, and consequently ordered the arm to be constantly supported in a sling, and kept perfectly quiet, and a bread and water poultice to be applied all over the joint. I also ordered half a grain of calomel, and five grains of rhubarb, to be taken every other night, with an occasional purge, and the greatest attention to be paid to the diet, according to the rules I have previously laid down.

"The plan of treatment, with very little variation, was pursued for a space of two years, when the disease might be considered as recovered from. There were, however, some slight variations made in the treatment, though the principles were the same, which will be noticed presently. For a considerable period, the joint appeared completely anchylosed, but subsequently it became evident that the bones had not united, and the sphere of motion has gradually increased. The os humeri of this arm was above an inch shorter than the other, and its head, though completely in its natural situation, was considerably reduced in size, being much less than it naturally should be. The glenoid cavity, however, was not destroyed, as its eyes were very perceptible from the wasting of the muscles. The appearance of the joint was such, that a superficial observer might have almost believed, if he did not know, that the joint had been diseased, that the humerus was dislocated: indeed a surgeon who examined it gave that opinion. Since the motion in the joint began to return, the muscles have also begun to increase in size.

"At one period the soft parts round the joint swelled considerably, and there was a great deal of tenderness. In consequence of this, leeches were occasionally applied, but never more than one or two at a time. Their application in this manner relieved the pain, and did not disorder the general health. After this, when the parts had got into a quiet condition, the surface of the joint was kept for several months in a state of soreness, by applying the tartar emetic ointment every morning; during this time, however, a poultice was applied every night, but in the day the shoulder was covered with flannel to promote external irritation. No other kind of medicine was given during the whole two years than what has been described, and this was continued the whole time: the form, however, was sometimes altered, but more to amuse parents than from any actual necessity. The diet, too, was sometimes varied a little, but the quantity was always regulated as attentively as possible."

Scrophulous Hip-Joint. *Pathology.* The progress of scrophula in this joint is so imperceptible that its ravages are often extensive before they are discovered to have commenced. Besides experiencing more or less constitutional derangement, the patient is at first a little lame and walks on his toe; he complains of being tired much sooner than he would if he were in health. After an uncertain space his lameness increases, and the affected limb is never advanced so far in progression as the other; in some instances it appears to be elongated; in others, shortened; but this entirely depends more on the position of the pelvis with respect to the spine. Lancinating pain is occasionally felt in the joint itself: in other parts of the limb, and particularly in the knee, the femoral and glutæal muscles emaciate; the thigh becomes evidently smaller; the nates appear widened and flattened: pressure on the joint behind the great trochanter, or in the groin, inflicts acute distress; and the bone around its external tuberosity feels thickened and enlarged. Not unfrequently the inguinal glands sustain scrophulous disorganization, and become the site of large abscesses. The extreme parts around the head of the thigh also undergo the same change, and sooner or later the articular capsule inflaming, suppurates and ulcerates, occasioning great functional derangement, destruction of parts, with excruciating pain. Cheselden relates two cases, in which the matter made its way through the acetabulum into the pelvic space.

"When matter," says Mr. L. p. 190, "does form in the cavity of the joint, it is often very suddenly, and it is always attended by a great accession of pain and general disorder. At this period the slightest motion of the joint often produces the most excruciating pain, which however is generally much relieved by the matter finding an exit. When the capsule is distended, there is usually much fulness in the groin and behind the great trochanter, and pressure on these parts occasions very great uneasiness. When matter collects in the articular cavity, from its distance from the surface, and from the thickness, great weight, and pressure of the superincumbent integuments, it often insinuates itself in various directions above and below the joint, before it makes an outlet, and this is frequently at a great distance from the joint, so that the matter cannot be discharged at one time, and other external suppurations take place, to give vent to the extravasated matter. These abscesses seldom close after they have discharged their contents, or, if they do, they soon re-open by fresh suppuration and ulceration; but they generally leave sinuses passing in various directions, yet all ultimately communicating with the joint, from which, there is a yellow serous fluid constantly discharged, and occasionally pieces of complete scrophulous matter, till the original disease in the bone is cured, or death terminates the case."

*Treatment.* Having instituted the usual course of general treatment, Mr. Lloyd enjoins the patient's being kept in a state of the most perfect rest; his being constantly confined to bed, and always lying in an horizontal position; his being incessantly retained in a straight posture with the trunk of the body prevented from inclining to either side, and having the heels placed as near as possible in the same line. He dissuades us from having recourse to local abstractions of blood by means of cupping-glasses or leeches, but acknowledges his having found counter-irritants to be sometimes very efficacious in preventing the formation of abscesses.

"Various modes of making counter-irritation," says he, p. 197, "have been proposed, such as the actual cautery, burning with moxa, issues, setons, scarifications, perpetual blisters, tartar-emetic ointment, &c. and most of them, no doubt, are sufficient to produce the necessary irritation. Issues, however, or the keeping a part of the external surface of the joint irritated and discharging, with the tartar-emetic ointment, I prefer. At the same time that these means are adopted, I have observed that, the efficacy of the treatment is much increased by enveloping the joint in a bread and water poultice. If an issue be decided upon, the preferable place for making it is just behind the great trochanter. If, after the issue is established, there is still a great deal of pain remaining, Mr. Crowther recommends the insertion of a seton in the groin: and it may be a useful practice; but in none of the cases I have met with, has it been necessary. I fully believe, that when counter-irritation is made in a judicious manner, at an early period of the disease, and if rest and proper constitutional treatment be employed, at the same time, we shall very seldom be troubled by the formation of abscesses, and, consequently, our patients will recover in a much shorter time. Nothing, however, can be more uncertain than the period of recovery, for we sometimes see cases, which, at first, have a most threatening appearance, recover in a few months; while, at other times, we see cases, that at first were not half so severe, almost as many years in recovery."

When a person, having diseased hip-joint, is first allowed to walk, he ought to use crutches, and be careful to save the affected limb. If anchylosis has taken place, which, however, seldom happens to scrophulous joints, such precautions will be less necessary. Mr. Lloyd has invented a splint which effectually obviates all motion of the joint, and restrains the undue actions of its muscles. It consists of a spring, which is firmly attached to the pelvis above the trochanter major, and to it is fixed a long splint, which is applied to the limb in the ordinary manner with or without an inner and outer short splint. It occasions no pressure that can possibly be injurious, nor does it create the least uneasiness. This section is concluded with several important cases, which we regret our limits oblige us to exclude.

Scrophulous Spine. Two kinds of spinal curvature—*angular* and *lateral*—depend on scrophulous disease. In the former there is always destruction of some portion of the vertebral column, and often, for a considerable time, progressive disorganization of bone, cartilage and ligaments: in the latter no destruction of parts, but merely an alteration of structure takes place. The vascularity of the bones, however, is increased; their earth is absorbed, and they become softer than natural. The ligaments also undergo some morbid changes, the connexions between the different bones are loosened and relaxed. When the curvature has existed long, the vertebral cancelli receive the scrophulous deposition, and ultimately, if the defect of constitution be not repaired, sustain the ravages of carious decay.

*Angular Curvature.* This generally occurs in the upper part of the dorsal vertebræ, but it may take place in any portion of the spine. It can never exist, even in the smallest degree, without some destruction of bone, which is necessarily a source of unabating irritation, and disturbance of the general health. It occasions a positive projection from the natural surface of the back. When two or three of the vertebræ are almost completely destroyed, and the adjoining ones sound, the projection is very direct. The angular is sometimes complicated with lateral curvature of the spine. The effects of this condition of the vertebræ are—communication of diseased actions to, or excitement of irritation in the spinal marrow and nerves connected with it; and the production of inflammatory manifestations, and abscesses in the contiguous soft parts.

Diseased action thus imparted to the spinal chord and the nerves is generally characterized by—constant and local pain, which is sometimes diffusive; paralysis, more or less, of the lower extremities, or of the vesical or anal sphincters; diminished power in the coats of the intestines and bladder; sense of sinking at the stomach; respiration slower than natural, with occasional interruptions; intermitting pulse; and wasting and flaccidity of the muscles of the extremities. Mere irritation about the medulla spinalis, or the origin of the spinal nerves, is distinguished by—diminished power in the lower extremities, accompanied by convulsive twitchings and irregular actions of some of the muscles, and sometimes with rigid spasmodic contractions of them; so that the foot and toes are drawn downwards, and the legs upwards and backwards, and immoveably bent on the thighs, together with a feeling of great tightness or constriction round the ankles; sense of coldness and shooting pains, with wasting of the muscles of these parts; lancinating pains in the chest; sense of tightness and uneasiness at the pit of the stomach; hurried respiration, with occasional convulsive actions of the diaphragm; quick agitated pulse; and all the symptoms of general debility and irritation.

Paralysis, in these cases, according to Mr. L. is not occasioned by any pressure of the diseased bones, but by the accumulation of lymph or matter in the spinal canal, or of some kind of fluid in the membranes of the spinal marrow.

"When abscesses form in these cases," he goes on to say, p. 228, "they generally originate in the soft parts interiorly, or about the anterior surfaces of the vertebræ, but sometimes in the external parts about the spinous processes. There frequently is no evidence by which it can be ascertained that the suppurative process is going on, until the matter makes its way to the surface of the body, and forms what is termed an abscess. The point at which it may first appear is quite uncertain, and it often is at a great distance from the seat of the primary disease. When the seat of the disease is in any of the lumbar or in any of the lower dorsal vertebræ, it is most common for the matter to first appear in one of the groins, either above or below Poupart's ligament, but there is no certain spot, and the matter often makes its way to the surface in the loins on one side of the lumbar vertebræ, sometimes by the side of, or above the anus, and indeed in various other situations. These abscesses generally go by the name of lumbar or psoas abscess. Nevertheless, the cause of their formation sometimes has no connexion with the lumbar vertebræ, as I have known this part of the spine perfectly sound, and the whole disease in the dorsal vertebræ. The nature of these abscesses is always readily discovered, as by applying the hand to the most prominent part. While the patient coughs, propulsion of the matter from within outwards is very distinctly felt. The quantity of matter that these abscesses contain is often enormous. I have seen five pints discharged at one time, and the discharge of two or three pints is very common."

When, from disease high up in the vertebræ, an abscess forms in the posterior mediastinum great danger is to be apprehended. The matter may insinuate itself in every direction, separate the pleuræ, produce ulceration of the diaphragm and peritoneum, and be diffused among the intestines, or the peritoneum remaining entire, it may detach that membrane from the diaphragm, and penetrate between the different layers of its processes, and by exciting peritoneal inflammation, accelerate the sufferer's death. Scrophulous matter, when confined to the mediastinum, and pressing on the lungs, causes very distressing difficulty of breathing. At last, it is discharged into the cavity of the thorax, and occasions a miserable death; or it makes its way externally, by ulceration on one side of the vertebræ, and is continually issuing through the aperture. Sometimes, after such abscesses have burst, the matter, when the patient coughs, will be propelled in a full stream to a considerable distance.

*Lateral Curvature.* This kind of spinal curvature depends, in most instances, on that particular alteration of structure in the vertebræ which is produced by scrophula in all other bones. Many authors refer its cause to the action of the muscles. Mr. L. allows that this may be the *immediate*, but not the *primary* cause, which he regards as proceeding from the state of the vertebræ themselves. When the disposition to the disease is formed, and scrophulous action has commenced in these bones, it increases their vascularity and softens their texture, relaxes their ligaments, loosens their connexions, induces atrophy of certain muscles, and weakens the spine; and, by the disbalancement of muscular function thus determined, irregular actions take place in the parts, and curvature ensues.

"If we have proof," adds Mr. Lloyd, "as I maintain we have, or if it be admitted that this state of the vertebræ exists, it is undeniable that the other symptoms or effects are what we would naturally expect to follow, for they are precisely what we witness in every other part of the body—that when a muscle ceases to be useful, or its action gives pain, it ceases to act at all, except when unnatural actions are produced, as spasms or convulsions."

According to Mr. L. the symptoms which often attend this affection of the spine clearly indicate that there is some other cause of local irritation than the mere curvature. They are sometimes as severe as those of the angular kind, and evidently arise from irritation of the spinal column and its nerves. Such cases are occasionally characterized by severe pain in the back; total or partial paralysis of the lower extremities; shooting pains along the intercostal nerves, and those of the lower limbs; twitching of the muscles; difficult or hurried respiration; paroxysmal convulsions of the diaphragm; involuntary discharge of fæces and urine. More frequently, however, the common symptoms are those of genuine debility, pain in the back and head, with other indications of nervousness and broken health.

"That these symptoms," says Mr. L. p. 238, "are not produced by the curvature is certain, because, in this affection, as in the angular curvature, all the symptoms of irritation may be removed, although the curvature remains as great as ever; but that they are produced by a morbid condition of the vertebræ, is at least probable, because, as the peculiar state of health which I maintain always precedes and attends such a morbid affection of the bones is removed, and, of course, at the same time, the disease in the vertebræ, all symptoms of irritation subside, so that the patient may continue for many years after, free from all inconvenience but what arises from his lamentable deformity."

There is much irregularity and uncertainty in the general symptoms which announce impending curvature of the spine, but, according to Mr. Lloyd's experience, there is a local symptom that invariably precedes its development. This is *the wasting of the muscles of the back*. It is commonly evinced in children by their stooping or bending their head more forward than is natural; or, when sitting, by their always raising their shoulder, and keeping their bodies bent forward; or by their constantly sitting or standing with one shoulder elevated above the other; or by their always lolling or leaning, when sitting down. The spinous processes, under such circumstances, are unusually prominent, and there is a deep depression on each side of them. This is more considerable on one side than the other, and is occasioned by the great and particular wasting of the muscles of the spine. Angular curvature in the slightest degree is detected immediately if it exists. In the lateral, the earliest symptoms are an alteration in the relative height of the shoulders and of the hips; a corresponding change in the shape of the chest, the spine taking on the form of an italic *S*, and the projection of one of the blade-bones, farther from the trunk than the other, partly owing to the thoracic deformity, but much more to a species of paralysis, or relaxation of the scapulary muscles.

*Treatment.*—The constitutional treatment of spinal curvature differs in no respect from that of scrofula in any of its forms. Among other local means the importance of rest and a reclining posture cannot be too impressively enjoined. It will secure the diseased parts from the irritation necessarily consequent on all muscular movements: it will preserve the body in an erect state, or support the parts superior to those vertebræ in which the destructive process is going on: it will contribute to avert deformity, and to diminish all those secondary effects so commonly attendant on this morbid condition. When the sick are permitted to escape this confinement, the duration of which will be determined by existing circumstances, it is advisable that, for some time, the back shall receive a certain degree of support. Mr. L's method of accomplishing this purpose will be subsequently described.

Next to quietude and a horizontal posture, is the adoption of some method by which counter-irritation near the part affected, and with as little disturbance to the patient as possible, can be unremittingly maintained. Issues or setons will accomplish this purpose; Mr. L. prefers the former, with the object of preventing irritation from being excited in the contiguous parts, or removing it if it already exists. He does not expect them to remove vertebral caries, or to induce reproduction of bone; these ends can only be gained by amendment of the general health.

When there is abscess in any way connected with the spine, he instantly establishes an issue on the side of the vertebra, opposite to that where the matter issues. He believes it is better to delay opening lumbar abscesses as long as practicable, for when the collection of matter is very great, it is often wholly absorbed. His mode of evacuating them is according to Mr. Abernethy's directions. A series of appropriate cases is adduced in confirmation of these practical rules; but we can only tell the reader they are excellent, and proceed.

For the amelioration or removal of lateral curvature, Mr. Lloyd recommends—attention to the general health; the reclining posture; issues, setons, perpetual blisters, and frictions with the tartar-emetic ointment, and stimulating liniments; and the patients being clothed from head to foot in flannel.

"When the curvature is great," says he, p. 261, "or there is indication of much disease, it is always of the utmost importance to keep the patient continually lying down, day and night; and, as it is very common for the vertebræ of the neck to partake of the affection of the rest of the spine, and for the head to be permanently drawn more forward to one side, it is equally important in placing the patient in an horizontal position, to keep the head in the same line with the rest of the body and on the same level; and for this reason, it is necessary for the patient always to lie without any pillow or bolster. If these points be not attended to, it will be in vain to attempt to remove the deformity, for without such attention, it will be impossible to preserve the muscles in that state of complete inaction which is essential to perfect recovery; or if the head be elevated above the level of the trunk, it will always be found, during sleep, more or less turned to that side, which must necessarily increase the deformity."

"To give the necessary support to the back when sitting down, it is very requisite to have a small chair, with a kind of crutch attached to each side of the chair, which shall press in the most gentle manner against the ribs in the axilla, the arms of course hanging over them. It is necessary too, for the crutches to be made, so that they can be elevated or depressed at pleasure, as it is sometimes, though but seldom, desirable to have one side more elevated than the other. The heads of the crutches too, should be made to turn round, so that if necessary, they may be applied as an equivalent to a back-board, or keep the shoulders back.

"When it is determined that a patient shall make use of exercise, I have invented an apparatus which affords the necessary support to the spine. Crutches, such as have been already described, should be used; but as there is no chair to rest them on, they are to be fixed to two pads, one above each hip, which must be confined to their situation by a slight spring behind, and by a soft leather strap before. These crutches are so constructed, they can be easily lengthened or shortened. They may be applied under the common dress without inconvenience, or they may be applied over it, in a great-coat or a cloak worn with them. When the vertebræ of the neck are affected, it becomes necessary to support the head. The apparatus that I consider the best for this purpose, is constructed on the principle of the old steel collars, which are supported on a pad to some part of the back, and connected to the crutches which support the spine. To the brim of the collar a lunated portion of steel is attached, behind and before, to each of which there is a strap, the one for the back of the head to rest on, the other to support the chin."

Several important cases are detailed by Mr. L. illustrative of this mode of treating lateral curvature. We transcribe one, p. 278, which is marked by interesting character.

"A young woman of fair complexion, with light hair and eyes, was first attacked when about 17 years old. At this time there were no symptoms of nervous irritation, and all that was observed was that the right shoulder grew out, and was considerably higher than the opposite one. She however, gradually became weaker and more delicate till she was nineteen years old, when she so completely lost the power, or rather the government of her legs, that she was unable to walk. She was also, as her mother informed me, extremely hysterical. She had been under the care of several medical men, some of whom treated her complaint as hysterical and others as rheumatic, and the true nature of her disease had not been at all suspected, nor had it at the period I first saw her, although, with the other very marked symptoms of spinal disease, she had had for nearly a twelvemonth incontinence of urine. When I was consulted about her, it was nearly four years after it was first observed that her shoulder grew out. I found her in the most horrible state of nervous irritation it is possible to conceive; her limbs were not paralytic, but they were so convulsively affected that she had no power over them, and if it was attempted to make her stand up, she was immediately drawn forwards with great violence. Her pulse was very quick; and her bowels were obstinately costive, not being open above once or twice a week. Her appetite was tolerably good. She complained of great pain in her loins, and the incontinence of urine still continued. I was also informed that she had frequent fits which were considered as hysterical. That all her symptoms were the effect of spinal irritation, appeared clear to me, and I therefore examined her spine, and found as complete an S as can be well imagined, and the greatest wasting of the muscles of the spine. I consequently ordered her to be immediately cupped on the back, and a large blister to be applied over the same part directly after, and to be kept open. I also directed her to lie constantly on her back, except while the blister was dressed, and at those times she was only turned on her side. To guard against her moving in the night, and to keep her legs quiet, her feet were confined to the bottom of the bed. The only medicines that were given her were aperients, to obviate the costive state of her bowels, and occasional doses of ether, in some camphor mixture, to relieve a continual sense of suffocation which she complained of. The good effect of this plan of treatment was almost immediate, as in less than a fortnight all the most violent and distressing symptoms had left her. Her bowels, too, were much more easily acted on; and she could sometimes retain her urine for nearly the whole day. To relate the details of this case, through the year and a half she continued a plain treatment founded on the same principles, would be tedious and uninteresting; I shall therefore merely add that, at the expiration of that time, she got up quite well, and with her back as straight as ever: notwithstanding, during the first twelve months after, she had occasional attacks of spinal irritation, but they were always relieved by rubbing in a little tartar-emetic ointment. It is worthy to be noticed, that this patient also had a scrophulous affection of the left breast; but subsequently she became quite well, and has been married for nearly a twelvemonth."

Pulmonary Scrophula. When the lungs take on diseased action, in a scrophulous state of the constitution, the first change they undergo is the deposition of cheesy matter into their parenchymatous structure. This operation goes on to form masses or tubercles that enlarge in size and increase in number to an indefinite extent. They are contained in cysts, which, according to Mr. L. consist entirely of condensated pulmonary substance. The tubercles themselves are certainly unorganized matter; for, instead of showing tendency to form vascular connexion with approximating parts, they act on them as extraneous bodies. The cysts, however, preserve vitality to the last, and their vessels can be readily injected. The lungs will often remain for a considerable time in this state, without experiencing any other effect than what arises from the mechanical action of the tubercles on their tubular texture. At last, by a species of slow inflammation, the substance of the lung, to an uncertain extent around the tubercle, becomes gradually consolidated.

" Sometimes," according to Mr. L's observation, p. 287, "the lung will undergo this change all round the tubercle, but at other times only on one side. When this alteration has taken place, the bronchia or tubular structure of the lung is obliterated, and its consistence is more like that of the liver than of the lungs. Sometimes, too, the lung gets into this state while the tubercles are of the smallest size; but when this is the case, they are generally exceedingly numerous. From what I have witnessed in dissection, I am disposed to believe, that even this change of structure may exist for a considerable time, without any further alteration; and that it may take place in different parts of the lung, and to a very great extent, at the same time. Under these circumstances the tubercles themselves undergo no change; and after this period, I believe, they are never enlarged; because, at parts where suppuration has actually come on, and therefore where the disease may be supposed to have existed longest, the tubercles appear rather smaller than larger. At length, however, more active inflammation is set up in the altered part of the lung surrounding the tubercle, suppuration takes place, and the third stage of the disease is established. At this period when suppuration has occurred, the tubercle will sometimes be broken down, and mixed with the common matter, but at other times it will remain entire; and it should be remembered that it is not the tubercle which suppurates, but the surrounding parts. The extent of the suppuration will vary much, but when it is connected with only one tubercle, it will be small, and form what is called a *vomica.* When, however, from the proximity or coalescence of several tubercles the consolidated part of the lung is great, it is common to have one large abscess form, but this does not preclude the contemporary formation of several smaller abscesses or vomicæ. Dissection seems to prove also, that though vomicæ may be at first distinct, several of them may afterwards, by enlarging, coalesce and constitute one large abscess. Although this may be the state of the disease in one lung, the opposite one may be in the first stage of the disease, or it may even be perfectly free from all morbid affection; and the same observation is sometimes applicable to another lobe of the same lung. It generally, however, happens, that there is a succession of small abscesses, and that while the contents of one are evacuating, another abscess is forming, and that in the same lung there will be tubercles just formed, tubercles with the lung immediately around inflamed and consolidated, and tubercles forming part of the contents of the vomicæ. The contents of vomicæ generally find their way into the bronchia, and are discharged by coughing; but sometimes there is no exit, as, the suppuration having been imperfect, the surrounding substance of the lung is thickened and consolidated. When a larger abscess forms it may be discharged in the same way; but it occasionally happens, that when it is situated in the upper part of the lungs, it will burst into one of the larger bronchial ramifications, fill the trachea with matter, and produce suffocation, and almost instant death."

From this descriptive view of tuberculous development in the lungs, which our readers will perceive is different in some respects from that given by Dr. Baillie, and other eminent pathologists, Mr. L. proceeds to sketch the symptoms of this diseased condition. His sketch is sufficiently faithful and perspicuous, but it is defective in delineating nothing more than the progress of tuberculous phthisis, terminating in ulceration and the discharge of purulent fluid from the lungs. We look in vain to find in it a clear diagnostic symptom or class of symptoms, by which we shall be able to ascertain whether this ulceration is the consequence of simple or specific inflammation—whether the expectorated matter is secreted in a scrophulous ulcer, or in an ulcer consequent to any other of the various forms which inflammatory excitement assumes: his words are—

"Although at an early period of the suppurative stage, the appearance of the matter will seldom enable us to judge accurately of the nature of the disease, still, at a more advanced period, it is an unerring index. It is impossible accurately to describe it, but I may observe that it seems as if composed of mucous, pus, and a curdy matter, of a whitish green, or yellow colour. Sometimes it is streaked with blood, but it seldom happens that this 's constantly the case."

Mr. L. does not discuss the treatment of pulmonary scrophula, but concludes this branch of his subject with an observation which we should be unwilling to overlook. From the nature of the disease, it appears to him that the only remedy from which there is any chance of deriving much benefit is counter-irritation, and that this should be made without producing additional disturbance to the general health. For this purpose he prefers blisters, and advises their being much larger than they are commonly made, which may be done without exciting any constitutional disorder. In regard to the possibility of tubercles being resolved or absorbed he is undecided; but seems inclined to think, with Dr. Cullen, "that Nature sometimes resolves and discusses tubercles which have been formed." We consider such a result as being of rare and difficult attainment, but not absolutely impracticable. We have already said as much in another part of this paper.

*Cardiac Scrophula.* Wiseman relates a case in which a scrophulous swelling, weighing two ounces, was attached to the apex of the heart. Mr. Lloyd met with the disease in a rabbit. In this case, there were three masses of scrophulous matter nearly as large as a pea in the substance of the left ventricle, and one of them projected into its cavity. They exactly resembled pulmonary tubercles, the animal's lungs were studded with tubercles, and several vomicæ had formed; its kidnies were in a similar state; a hydatid was situated among the flexor muscles of its thigh, and another between the scapula and trunk. They were as large as a hen's egg, and contained numerous smaller ones. The scapulary hydatid was at one time taken for an aneurismal tumour. In this case, the heart's action was very violent, extreme emaciation ensued, and the creature had severe fits before it died. We are told by Portal that the heart has been found in a state of suppuration in phthisical and marasmal subjects.

HEPATIC SCROPHULA. Scrophulous tubercles in the liver are encysted in the same manner as in the lungs, and distinct from the substance of the gland, with which, however, they appear to be more closely connected. Merat* states that, in most instances, he has found them destitute of a membranous envelope. When affected with scrophula, the surface of the organ is generally smooth; at other times, it is uneven from the projection of tubercles. Hepatic tubercles do not so frequently go into the ulcerative state as those in other parts; but we are taught by Dr. Baillie to believe that they do suppurate. Mr. Lloyd has twice seen abscesses in the liver containing scrophulous matter. By the result of extensive necrotomical research, Portal† is satisfied that multitudes of the young, and many of all ages, perish from the effects of scrophulous engorgement of the liver, we shall epitomize one or two of the important observations by which this doctrine of his is confirmed.

1. " A young man, whose whole person was jaundiced, and neck covered with scrophulous tumours, died in a state of extreme emaciation, after having experienced very severe and frequent colic pains, diarrhœa, nausea, and hectic fever. On dissection, the brain was found uncommonly fine; the lungs adhered to the pleura in different places; their substance was more solid than natural, and the right superior lobe indurated; the liver was greatly enlarged, condensated, and hardened, especially the anterior margin of its left lobe; it weighed nine pounds; tubercles, some of which were as large as a hen's egg, studded its surface; these, like so many knobs, contained substances more or less concrete, whitish, greyish, reddish, some steatomatous, others atheromatous, others melicerous; in the internal structure of the gland were many tubercles, varying from the size of a millet-seed to that of a pullet's egg. The small ones were red as if inflamed; the rest contained a fluid resembling granulary whitish pus; in one word, the liver had undergone the same changes as the lungs do in scrophulous phthisis: the chyle-duct was much contracted in diameter, and its duodenal extremity compressed by a glandular body, similar to those found in the liver; the mesentery contained several steatomatous concretions; and the bladder was much indurated.

2. " A child, during dentition, became diseased, emaciated, comatose, and died. On its neck had long been a cluster of gangliformed tumours of different sizes; some of these were red, others somewhat softened; the right hypochondrium was tumid, the false ribs elevated, and the liver greatly enlarged; the inferior extremities became œdematous, the rest of the body bloated; the skin squamous and dry.—Serum, in considerable quantity, had been effused into the cerebral ventricles, between the cranium and dura mater, and

---

* Dictionnaire des Sciences Medicales, tome xvi. 1816.

† Observations sur la Nature et le Treatment des Maladies du Foie, a Paris, 1813.

into the vertebral canal; the encephalic textures were unchanged, the lungs and heart healthy; the liver was exceedingly voluminous, indurated, and covered with tubercles, some of which contained a dense substance resembling coagulated white of an egg; they were soft and greyish; the mesenteric glands were distended and full of similar tubercles; and there was an infinite number of concrete bodies, of the size of a small pea, interspersed between the coats of the stomach and of the intestinal canal.

3. " Madame C. had engorgement of the cervical glands in her youth; her spine was slightly curved, her constitution feeble, and excessively nervous; at puberty, her health improved; she afterwards married and had children; in her thirty-fifth year she suffered from mammary congestion and irregular menstruation; pains in the uterus; the os tincæ was found to be engorged; colic spasms supervened; the patient became jaundiced and voided red lateritious urine; she had frequent hiccup, dry cough, copious and constant expectoration of whitish puriform matter; fever was kindled, with acute pain in the right side; medicine produced no relief, and the patient died. Inspection of the body exposed incipient cancerous ulceration of the uterus; the liver, uncommonly enlarged, was full of whitish tubercles, resembling coagulated white of an egg, and contained suppurating ulcers, (des foyers de suppuration,) and the left lung was gorged with purulent sanies. There can be no doubt, concludes the venerable reporter, that a scrophulous affection was the cause of this lady's death, and that she expired under a triple malady—uterine cancer, hepatic phthisis, and scrophulous pulmonary consumption."

Pancreatic Scrophula. Mr. Lloyd has never seen, in the pancreas, any deposition similar to scrophulous tubercles; but he met with an instance of pancreatic abscess containing two ounces of fluid, very like what usually results from scrophulous suppuration. Wiseman mentions a case of the same nature, in which the abscess contained a pint of matter. Suppuration of the pancreas is described by Portal, as being frequently so considerable, that its substance (son tissu) is almost entirely destroyed. The pus formed under such circumstances, exhales an intolerable fetor, and is sometimes of a greenish colour, sometimes greyish-white. When the purulent secretion is consecutive to scrophulous suppuration, to which the pancreas is as subject as the salivary glands, the fluid is whitish and grumous. Pancreatic abscesses, according to his observation in numerous instances, are contained in a sac, cyst, or membranous envelope, formed of the cellular membrane with which the organ is invested. He found an abscess of this kind distended with two pounds of pus, and he has known others insinuate themselves through the meso-colon and pour their contents into the abdominal cavity. He regards ulceration and cancer of the pancreas as frequently superpervening on scrophulous enlargement and scirrhus of that viscus.

SCROPHULOUS SPLEEN. Scrophulous tubercles, according to Mr. L. form in this organ in the same manner as in the lungs, but they generally acquire a larger size, sometimes, however, they are numerous, very small, and diffused through the whole gland. Dr. Baillie believes they are much more rarely formed in the spleen, than in the lungs, but they are precisely of the same nature in both.

GASTRIC AND INTESTINAL SCROPHULA. We shall pass from what Mr. Lloyd has written on this branch of the subject and copy Dr. Monro's description of scrophula as it appears in the alimentary canal.

"Scrophula of the alimentary canal is a disease which, though frequent, has not been described with sufficient accuracy. When the stomach and intestines become the seat of scrophula, all the coats of the part affected attain an unnatural thickness. I have seen the coats of the stomach half an inch thick, and instead of being heavier and harder, and indurated, as from scirrhus, they become softer, and more of a spongy consistence than natural. There is another peculiarity in this organic derangement, which distinguishes it from cancer; it is not limited to the cardia or pylorus, but affects the whole of the stomach; and, instead of being limited to a certain portion of the intestines, affects the whole intestinal canal. Upon making a section of the diseased intestines, we cut through a substance of an uniform consistence, which somewhat resembles the white part of the skin of an orange, through which a great many blood-vessels are distributed. The coats of the diseased part are rarely of an uniform thickness, being generally thicker towards the right side of the stomach, where I have seen them half an inch in thickness. In some cases, we observe small conical swellings growing from the villous coat of the stomach, and upon the same coat there are generally patches of a florid red colour; the stomach always contains a quantity of a very viscid mucus. A very peculiar species of ulceration follows the unnatural thickening of the coats of the part affected by scrofula, and which was, I believe, first described by Dr. Carmichael Smyth, in the second volume of the London Medical Communications. The size of the ulcer varies from that of a six-pence to that of a crown-piece, its form commonly circular or oval: the erosion of the villous coat is more extensive than that of the muscular; the perforation or opening through the peritoneal coat is much the smallest, and seems produced by sudden rupture. The edges of the ulcer are found well defined, somewhat thickened, rounded, and sometimes hard, a proof of its long standing. The disease is confined to the villous and muscular coats of the stomach, whereas the peritoneal is always free from disease.

The kidney is very subject to scrophulous action. Sometimes the extent of scrophulous suppuration is such that the whole substance of the gland becomes destroyed, and nothing but a bag remains. We shall pass over the section on scrophulous affections of the brain, and also that on scrophulous ophthalmia, with the exception of some short notice of Mr. Lloyd's description of the ophthalmia so long prevalent in Christ's Hospital.

This disease was evidently of a purulent kind, but commenced very gradually, and in both eyes at the same time. Slight inflammation of the palpebral conjunctiva ushered in the attack, attended by a sense of weakness in the eye, and a low degree of intolerantia lucis. This state would often exist for several months, though gradually increasing, till, from some accidental cause, as a cold, blow, or introduction of some foreign substance, a sudden and great increase of inflammation would take place, during which the tunica conjunctiva would become so much thickened or granulated as to form distinct folds at that part where it begins to pass from the eye to the eyelids. This stage of the disease was sometimes painful, sometimes not—and the same might be said of the sensibility to light—and of the general health. Except in bad cases, there was little acceleration of pulse, and no particular whiteness of the tongue. This stage often existed but for a few days, and seldom continued more than a fortnight, except where there was constitutional disturbance. With the cessation of the acute inflammation the third stage might be said to commence. This was distinguished by a sense of weakness in the eyes, and dimness of sight; but particularly by the immensely thickened state of the conjunctiva, and the discharge of a muco-purulent matter, which often lay in masses on the surface of the granulations, or between the folds of the diseased conjunctiva.

In the treatment of this disease, there was nothing to be done during the first stage, or when there was only an appearance of slight inflammation of the conjunctiva palpebrarum, but to wash the eyes occasionally with some soothing or very mild astringent lotion—to anoint the edges of the eye-lids with a mild ointment at bed time—to keep the bowels regular—and to avoid all sources of irritation. Our author, however, confesses that neither this nor any other treatment ever arrested the progress of the disease effectually. When the eye became once affected it went through the three stages abovementioned, "however liberally emetics, bleeding, and blisters were made use of." Much may be done to mitigate the violence of the disease, however, by judicious treatment in this and other stages of the complaint.

In the second stage, the treatment consisted in bleeding according to circumstances—blisters—tepid fomentations—mild astringent ointments—purgatives. When the growth of granulations or new parts was excessive, and the eye-lids had become everted, the argenti nitras, or even the kali purum was the most efficacious application. Instead of giving much pain, as might be expected, there was generally an immediate wasting of the granulations. Care must be taken, of course, that the caustic does not come in contact with the cornea. Mr. Lloyd much prefers this mode to excision; for in the latter case, new granulations were speedily produced. In the third stage, when the inflammation has ceased, and the parts are in a quiet indolent state, the principal part of the treatment consists in the removal of the new granulations, or newly formed surface of the conjunctiva, which may be effected in three ways—first, by the mere action of the absorbents, assisted by the judicious use of stimuli—secondly, by the caustic, as was stated before—thirdly, by excision with the knife or scissors. Of these Mr. Lloyd has found the first mode the most efficacious, except in those cases where the formation of new matter had been so excessive as to produce mechanical injury. Of all stimuli he prefers the saturated solution of argenti nitras, as being the most expeditious in its effects, and least painful in operation. We may, however, commence with it a little weaker, and gradually increase its strength. The application should be made every day, or every other day. The efficacy of this treatment is proved by the results at Christ's Hospital, where not a single unfortunate case happened among the boys, though several hundred of them were attacked by the disease. Not even the slightest shade of opacity remained in more than five or six cases out of that number.

After the ample analysis which we have presented of Mr. Lloyd's work, it is hardly necessary to state any opinion on its merits. We shall therefore conclude with a very laconic judgment—it *deserved* the prize awarded by the College.

